# The Neural Basis of Salt Perception: A Focus on Potassium Chloride as a Sodium Alternative

**DOI:** 10.3390/life15020207

**Published:** 2025-01-30

**Authors:** Emilia Iannilli, Raffaela Fürer, Antje Welge-Lüssen, Thomas Hummel

**Affiliations:** 1 Department of Psychology, University of Graz, 8010 Graz, Austria; 2 Smell & Taste Clinic, Department of Otorhinolaryngology, Technische Universität Dresden, 01307 Dresden, Germany; 3 Department of Otorhinolaryngology, University Hospital Basel, 4031 Basel, Switzerland

**Keywords:** taste substitution, salt alternatives, spatiotemporal dynamic of taste, microstate segmentation, source analysis

## Abstract

Excessive dietary sodium intake is a major risk factor for hypertension, prompting interest in potassium chloride (KCl) as a sodium chloride (NaCl) alternative. While KCl preserves saltiness, its neural processing compared to NaCl remains underexplored. This study investigates the neural correlates of taste perception for NaCl, KCl, and their mixture using gustatory event-related potentials (ERPs) in a sample of 28 healthy young adults. Participants rated the intensity, saltiness, and pleasantness of the stimuli, which were matched for iso-intensity and iso-pleasantness. High-density EEG data revealed distinct microstate patterns associated with each condition, particularly in the later stages of processing, which align with the endogenous phases of taste perception. Source localization identified the insula and opercular regions as primary sites for gustatory processing, with specific differences in activation patterns between NaCl and KCl. These findings suggest that while KCl elicits comparable behavioral responses to NaCl, its neural representation involves unique processes that may reflect its distinct chemical properties. This study advances our understanding of the neural dynamics of salt taste perception, providing insights into the potential use of KCl as a potentially healthier alternative in dietary interventions.

## 1. Introduction

Hypertension, a leading global health concern, is rapidly on the rise, with an estimated 1.5 billion people projected to be affected by 2025 [1]. This condition is a significant risk factor for cardiovascular diseases and strokes, leading to substantial healthcare costs and societal burden [2]. Among the factors contributing to the development of hypertension, dietary sodium chloride (NaCl) intake has been closely linked. Reduced consumption of NaCl, commonly referred to as table salt, has been associated with reduced blood pressure [3,4,5] and the subsequent lower risk of cardiovascular events [6], stroke [7,8], and left ventricular hypertrophy [9,10]. The World Health Organization (WHO) recommends a daily mean sodium intake (g/day) of less than 5 g, which is significantly lower than the daily mean sodium intake registered in the global average consumption of 9–12 g/day [11]. These recommendations have led to various public health initiatives aimed at reducing NaCl intake. For example, the Croatian Hypertension League in collaboration with the Ministry of Agriculture and the Croatian Agency for Food and Agriculture in 2015 introduced a regulation to limit the salt content in baked bread to 1.4% and they formed an agreement with the meat industry to reduce the salt content in their products by 25%. In a recent analysis of the data in March of 2023 they found that salt intake had reduced from 11.6 g per day in 2015 to 10 g by 2021; parallelly, they recorded a mean blood pressure reduction of 1.9 mmHg at the population level, suggesting a positive effect of the regulation on the salt intake [11]. As part of these interventions, the substitution of NaCl with potassium chloride (KCl) has been proposed, as KCl provides a similar salty taste without the detrimental effects of sodium on cardiovascular health.

The way we perceive taste is influenced by the interaction of taste-active compounds with their specific receptors located in the taste buds. Recent research has identified receptors linked to five fundamental tastes, which can be categorized into two main types. G protein-coupled receptors (GPCRs) are primarily associated with the detection of sweet, bitter, and umami flavors [12], whereas the sensations of saltiness and sourness are mediated through certain receptors or ion channels, such as epithelial sodium channels (ENaCs) and transient receptor potential vanilloid type 1 (TRPV1) [13,14]. Generally, ENaC functions as a specific receptor for sodium ions (Na+), while TRPV1 is responsive to not only Na+ but also other cations like potassium (K+). The accumulation of these cations within taste receptor cells can lead to depolarization of cell membranes, triggering changes in intracellular calcium levels that result in the transmission of nerve signals and the perception of salty taste. However, the salt taste transduction mechanism is complex and is still under investigation. For instance, it has been observed that other minerals such as potassium, calcium, and magnesium, along with sodium (or their respective salts), may also activate sweet, bitter, and umami receptors, thus enhancing the perception of various taste qualities [15,16]. This makes the direct substitution of NaCl with KCl somewhat difficult due to the bitter/sour aftertaste that some people perceived when tasting KCl [17]. However, psychophysical studies, such as those by [18] Li et al., demonstrate that partial substitution of NaCl with KCl in food preparations can maintain palatability; see also [19,20].

Nevertheless, several studies suggest that increasing dietary potassium intake may help lower blood pressure in both hypertensive and normotensive individuals [21,22,23]. Despite these promising findings, there remains a crucial gap in our understanding of how these salt substitutes are processed in the central nervous system (CNS). Current research on salt perception has primarily focused on behavioral and psychophysical outcomes, often driven by the food industry. However, little is known about the neural mechanisms underlying the perception of salty taste, particularly when comparing NaCl and KCl. This gap is significant, as understanding the brain’s processing of these stimuli could provide deeper insights into taste perception, hedonics, and potential differences in how NaCl and KCl are perceived and evaluated by the gustatory system.

Event-related potentials (ERPs) in electroencephalography (EEG), a non-invasive method for recording brain activity in response to specific sensory stimuli, offer a powerful tool to investigate the neural correlates. ERPs have been widely used to study sensory processing in the auditory and visual domains, and their application in gustatory research is emerging as a promising approach to explore how the brain processes different taste stimuli [24,25,26]. Moreover, when ERPs are registered with high-density channel electrodes, they can provide accurate and reliable localization of the respective brain regions of interest, in addition to precise temporal information about neural activity [27,28,29,30].

In the present study, we investigate the neural activation elicited by different savory conditions using high-density gustatory event-related potentials (ERPs) with one of the state-of-the-art setups [31,32]. Specifically, we aim to compare the neural responses to KCl and NaCl and a mixture of them to study the neural correlate and palatability as function of the sodium component, the element associated with adverse cardiovascular outcomes. Our goal is to characterize the time series of the gERPs for NaCl and KCl in the typical initial 1000 ms of the brain process and to explore potential differences and similarities in its processing.

This study addresses an important gap in the literature by characterizing the neural dynamics of KCl and NaCl perception, contributing to the broader discussion on salt substitutes and their implications for public health. Ultimately, we conclude that while KCl may evoke similar gustatory responses to NaCl, its distinct neural processing may have implications for its use as a widespread NaCl substitute.

## 2. Materials and Methods

### 2.1. Participants

A total of 28 healthy, right-handed volunteers were included in the study: 15 women and 13 men aged between 20 and 34 years, with mean age ± sd = 25 ± 2.8 years. The health status was ascertained with a detailed medical history. Exclusion criteria included metabolic disorders (like diabetes and renal diseases), smokers, and pregnant and breastfeeding women. The gustatory and olfactory functions were tested, respectively, with regional gustatory testing using taste strips (group score: mean ± standard deviation = 14.5 ± 1.2) [33] as well as taste sprays (score 4/4) [34], and the Sniffin’ Sticks Screening Odor Identification test (group score: mean ± sd = 14.6 ± 1.1) [35]. All subjects included in this study had test scores within the normal range. Participants were informed about the experiment and gave written consent for anonymous personal data processing.

### 2.2. Gustatory Event-Related Potentials (ERPs) and Study Design

The brain signals measured after a gustatory stimulus in EEG are called gustatory event-related potentials (gERPs). To achieve accurate results, the onset of the stimulus must exhibit well-defined quasi-delta-function characteristics in both space and time (event) [35]. A pure taste event, without spurious sensations, such as mechano-, somatosensory, or thermal stimuli, requires a specialized delivery system. In our experiment, we utilized a computer-controlled oral stimulator that accurately delivers pulses of a liquid solution containing tastants at a controlled temperature with masked mechano- and somatosensory sensation [31]; detailed procedures with materials and methods are described in [32].

The psychophysics and EEG data were collected in two sessions on separate days. During the first visit, the participants underwent psychophysical testing to ascertain normal gustatory functions and a mock test of the gERP acquisition, where they became acquainted with the procedures during the investigation. The first visit lasted ca. 30 min. During the second session, we recorded the ERPs data synchronously with a gustatory stimulation, where a constant liquid flow irrigated the subject’s protruded tongue. Liquids were collected in a bowl positioned under the volunteer’s chin. For each savory condition, the volunteer had to rate the intensity on a visual analog scale from zero (not perceived) to 10 (extremely intense). A white acoustic noise delivered through headphones shielded the subject from surrounding possible noises. The second session lasted about 2 h.

### 2.3. Stimuli

In the first session of the study, five stimulus conditions were used. They were based on two different liquid stimuli of sodium chloride (NaCl) and potassium chloride (KCl), and their combination was achieved in different percentages. The concentration for NaCl (295 mM) and KCl (352 mM) was set using a psychophysical pilot study to match their intensities at an iso-value. Other conditions were a mixture of both with three relative percentages: 50% NaCl and 50% KCl, 20% NaCl and 50% KCl, and 20% NaCl and 70% KCl (volume percentage). The solutions were prepared in water for injection (Aqua ad injectabilia, Braun, Hessen, Germany). The participants were thoroughly acquainted with the experimental setup. The tastants were delivered using a computer-controlled pump system (gustometer, Burghart GU002–variant GM04; Burghart Messtechnik, Holm, Germany) in a pulse design within a continuous flow of neutral liquid (Aqua ad injectabilia, Braun, Hessen, Germany). With this device, the tongue was constantly stimulated and at a constant body temperature to mask somatosensory and temperature effects. The gustometer has been positively tested to induce gustatory ERPs [31]. The taste-pulse duration was 250 ms with a volume of 100 µL per pulse. The interstimulus interval (ISI) was jittered from 18 to 22 s (mean ISI 20 s) to reduce expectation effects.

In the first session, the participants had to rate the intensity (0 to 10), saltiness (0 to 10), and pleasantness (−5 = very unpleasant, +5 = very pleasant) on a continuous visual analogical scale presented on a screen and perform a simple tracking task to ensure alertness.

In the second session, the EEG acquisition took place. As final stimuli, we used three different conditions: NaCl (295 mM), KCl (352 mM), and the 50% mixture of the two in volume percentage. The choice was made based on the rating results obtained by the first session, where the group of the subjects evaluated the solutions as iso-intense (F[2,28] = 0.735; *p* = 0.488), iso-pleasant (F[2,28] = 0.278; *p* = 0.759), and with the same saltiness level (F[2,28] = 0.988; *p* = 0.385).

This study was performed in accordance with the Helsinki Declaration, and the local ethical committee approved it (EK 286112008).

### 2.4. EEG Acquisition and Data Pre-Processing

The EEG data were recorded using a 128-channel EEG system (Biosemi; electrodes: Ag-AgCl active electrodes, BioSemi; 10/20 BioSemi–CAP; Amsterdam, The Netherlands). Eight additional external electrodes recorded four vertical and horizontal electro-oculographic signals to control for eye artifacts. Each channel was amplified (BioSemi ActiveTwo AD-box) and sent via a single optical fiber to a USB2 receiver (BioSemi) connected to a PC. Data were registered and stored using BioSemi ActiveView (v.605) acquisition software. We used electrode gel (Signa gel; Parker Laboratories, Inc., Fairfield, NJ, USA) to maximize the conductivity and reduce electrode impedance. The taste event trigger was synchronized in parallel with the EEG signal.

A white noise source was applied via headphones to minimize the surrounding sounds. Blinking or sleeping/vigilance issues were controlled through a tracking task that the subject performed with the help of a computer mouse. The subjects were asked not to eat, smoke, or drink anything but water for 1 h before the EEG sessions.

We used Cartool software v.4.11 [36] for EEG data analysis. Signal pre-processing included baseline correction (500 ms before stimulus onset), a 15 Hz low-pass filter, a 0.1 Hz high-pass filter, and a 50 Hz notch filter. For each subject in each condition, data were epoched after manual artifact rejection and interpolation of bad electrodes. The EEG reference was set to the signal average. Before analysis, each subject’s data were anonymized and assigned to the relative group. A grand average for each condition was computed [37].

### 2.5. EEG Topographical Analysis and Source Localization

The core idea of topographic analysis [38] suggests that scalp topographies maintain stable configurations over time instead of shifting randomly, reflecting underlying neuronal processing. A randomization robust statistical test can identify stable scalp topographies, which are also called functional microstates [39]. Our analysis defined the optimal number of functional microstates based on the grand average for each taste condition using k-means spatial cluster analysis. Cartool has six implemented criteria that are reliable for EEG data (Gamma, Silhuettes, Davies and Bouldin; Point-Biserial; Dunn and Krzanowski-Lai Index) [40]; then, a meta-criterion, defined as the median of all optimal numbers of clusters across all criteria, individuates the optimal number of clusters [41]. Cluster maps that correlated more than 85% were merged, and segments less than or equal to 12 time frames (TFs) (24 ms) were rejected. The defined stable maps’ topography was then fitted back to each subject to validate the segmentation procedure [38].

The localization of the EEG sources in high-spatial-resolution brain structure was achieved by co-registering the EEG electrodes placed on the skin with an MNI template (Montreal Neurological Institute) with predefined translation parameters to match the two spaces. The coordinates of the localized areas were converted into Talairach space, which is the reference framework utilized by the brain atlas [42]. As a head model, we used a local spherical model with anatomical constraints (LSMAC) with space solution constrained to the gray matter for a total of 5000 solution points. A local autoregressive average (LAURA) [43] algorithm was applied to estimate the inverse solutions.

### 2.6. Demographics and Behavioral Data Analysis

The demographic and psychophysical data were analyzed by means of IBM SPSS v21 (IBM, Ehningen, Germany). We used a one-way repeated measures ANOVA to evaluate the intensity, pleasantness, and saltiness of the taste conditions.

## 3. Results

### 3.1. First Lab Session—Stimuli Ratings

**Figure 1:**
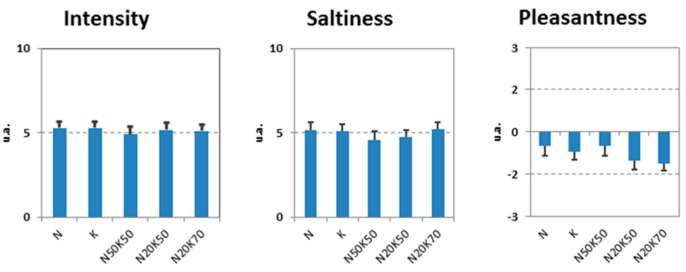
Intensity, saltiness, and pleasantness ratings for the 5 solutions in the first session. The group evaluated NaCl (N), KCl/K, and the mixture (N50K50), a 50% mixture of N and K, iso-intense, iso-pleasant, and with an indistinguishable level of saltiness. N: 295 mM NaCl; K: 352 mM KCl; N50K50: 50% of total volume constituted by N and 50% by K.

Mean values and standard error of Intensity, Saltiness and Pleasantness of the five combinations of NaCl and KCl are represented in Figure 1. By means of one-way repeated measures ANOVA, it was ascertained that the volunteers evaluated the stimuli (N, K, and N50K50) as iso-intense (intensity N [mean ± sd] = 5.3 ± 2.0; intensity K [mean ± sd] = 5.3 ± 2.1; intensity N50K50 [mean ± sd] = 4.9 ± 2.5; Wilk’s lambda = 0.950; F[2,28] = 0.735; *p* = 0.488), iso-pleasant (pleasantness NaCl [mean± sd] = −0.5 ± 1.9; pleasantness KCl [mean ± sd] = 0.7 ± 1.4; pleasantness N50K50 [mean ± sd] = −0.5 ± 1.8; Wilk’s lambda = 0.980; F[2,28] = 0.278; *p* = 0.759), and with the same saltiness level (saltiness N [mean ± sd] = 5.2 ± 2.3; saltiness K [mean ± sd] = 5.1 ± 1.9; saltiness N50K50 [mean ± sd] = 4.6 ± 2.6; Wilk’s lambda = 0.934; F[2,28] = 0.988; *p* = 0.385).

### 3.2. Gustatory ERPs

The ERPs were averaged after discarding muscular or blinking artifacts. The Supplementary Materials (Figure S1) include butterfly plots showing the grand mean, using as reference the average among the electrodes after artifact rejection and electrode interpolation on the group of subjects for each condition, with K on the top, N50K50 in the center, and N at the bottom.

### 3.3. Microstates Segmentation

**Figure 2:**
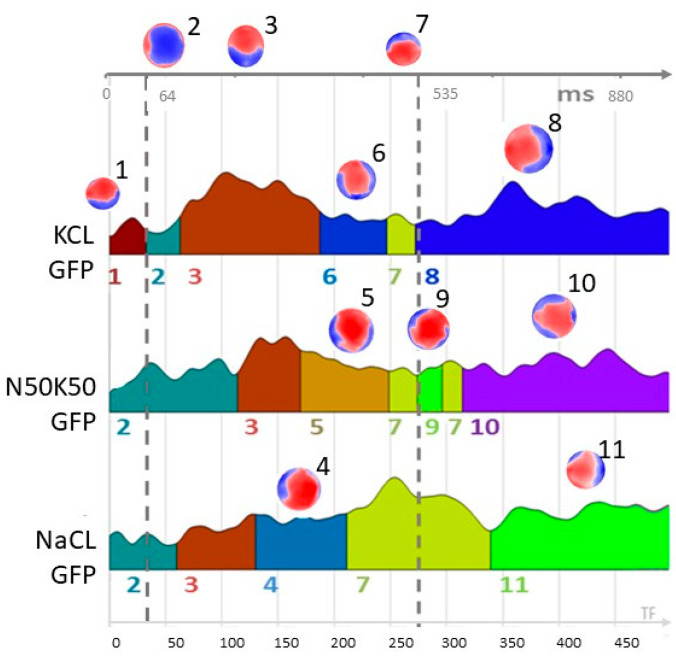
Microstate segmentation. The segmentation for the three conditions identified 11 stable maps (topographies), with positive voltage shown in red and negative voltage in blue, each coded by numbers. The duration of each microstate is color-coded with the corresponding map number on the global field power (GFP) of the ERPs grand average for each condition. Same color and same number indicates that the segment is occupied by the same microstate (Map). For example, the duration of Map 2 (microstate 2) is represented by the extension of turquoise color under the GFP curve for each condition. The x-axis (bottom and top of the graph) displays the temporal variable in both time frame (TF) and milliseconds (ms). It is important to emphasize that, considering the spatial correlation between Map 5 and Map 6 (r = 0.62), as well as that between Map 5 and Map 4 (r = 0.75), the time series of the maps remains consistent across the three stimuli within the time range of 64 ms to 535 ms (time range between the dotted lines). Specifically, this sequence follows the order of Map 2, then Map 3, followed by Map 5, and concluding with Map 7.

Our segmentation results show eleven stable topographic maps for the three conditions (Figure 2). The initial time course of the segmentation, including Map 2 and Map 3, is similar for the three conditions up to ca. 300 ms. At this point, a differentiation of maps is detected: NaCl condition is represented by Map 4, the mixture condition is represented by Map 3 followed by Map 5, and the KCl condition is represented by Map 3 followed by Map 6. The unique maps per condition are as follows: Map 1 and Map 8 for KCl, Map 9 and Map 10 for the mixture condition, and Map 11 for NaCl.

**Figure 3:**
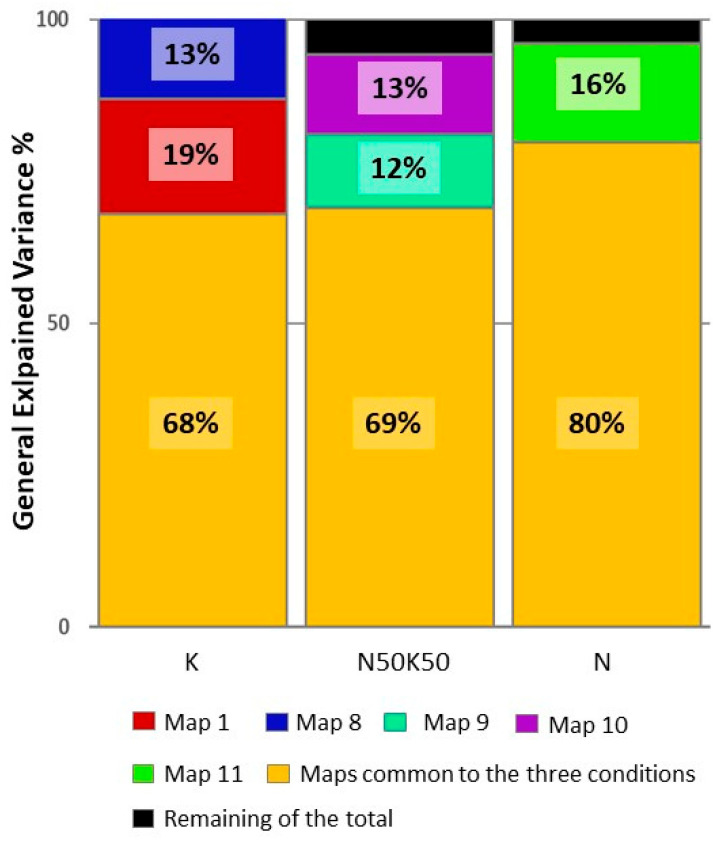
Mean general explained variance (GEV) calculated by averaging the GEV for each map derived from fitting data at the single-subject level for each condition. The 11 topographies identified through microstate segmentation were fitted back to the single-subject ERP-EEG conditions and averaged for each map in each condition. The maps that were common across conditions—Maps 2, 3, 5, and 7—accounted for the majority of the explained variance, with values of 68%, 69%, and 80% for K, the mixture (N50K50), and N (indicated in orange), respectively. When incorporating the respective unique maps, the mean GEV exceeded 90% of the total GEV for each condition, confirming the segmentation obtained at the group level. For K, Map 1 contributed an additional 19%, while Map 8 contributed an additional 16%. For N50K50, Map 9 contributed an additional 12%, and Map 10 an additional 13%. Finally, for N, Map 11 contributed an additional 16% to the GEV.

We fitted the identified maps to each epoch at the single-subject level to validate the segmentation obtained at the group level. For the purpose of fitting back, and because we observed a high correlation between Map 5 and Map 6 (r = 0.62), as well as between Map 5 and Map 4 (r = 0.75), suggesting a strong dependence among those maps not captured by the segmentation process, we used Map 5 as a substitute for both Map 4 and Map 6. Due to our prior consideration, we can say that the time range between 64 and 535 ms is represented by the same maps in the same order, regardless of the conditions. The common time series begins with Map 2, followed by Map 3, then Map 5, and concludes with Map 7. This consistent sequence of microstates suggests that they are generated by similar neuronal circuitry, irrespective of the taste conditions. Finally, we extracted each map’s general explained variance (GEV) at the single-subject level. The average GEV obtained from fitting back to each ERP-EEG subject is reported in Figure 3.

Based on the validation at the single-subject level, we identified five stable microstates that were uniquely present for the three conditions. For KCl, Map 1 occurred at the beginning, while Map 8 appeared in the last time frame of the epoch. For the mixture condition, Map 9 started after 534 ms and lasted approximately 32 ms, followed by Map 10, which occurred in the final epoch. Finally, for NaCl, Map 11 was present in the last time frame of the epoch. We will further estimate the brain sources associated with these specific microstates.

### 3.4. Sources Localization on the Microstates Specific for Each Condition

We computed the brain source responsible for the microstates Map 1, Map 8, Map 9, Map 10, and Map 11. We adopted the assumption of the spatiotemporal dipole model that dipole positions are unknown but fixed through time [44]. The estimated current density of the solution space was thresholded at the maximum activity for each topography, and we extracted the coordinate associated with the solution point identified by the maximal current density.

**Figure 4:**
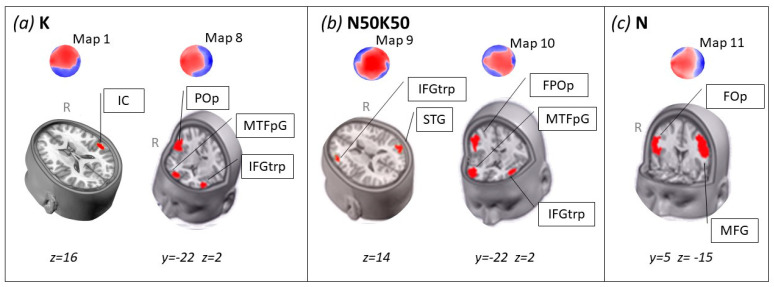
Source localization of the specific microstates for each condition. (**a**) K is distinctly represented by Map 1 and Map 8; (**b**) the mixture, N50K50, from Map 9 and Map 10; and (**c**) N from Map 11. IC: insular cortex; POp: parietal operculum; MTFpG: medial transverse frontopolar gyrus; IFGtrp: inferior frontal gyrus triangular part; STG: superior temporal gyrus; FPOp: frontoparietal operculum; FOp: frontal operculum; MFG: medial frontal gyrus; R: right hemisphere. For the axial and coronal cuts, the z and y Talairach coordinates (mm) are indicated at the bottom of each frame, respectively. For the maps red indicate positive voltage and blue negative.

Table 1 presents the estimated brain sources from specific microstates for each condition, along with their Talairach coordinates of maximum activity and the definition of the solution point in the cubic matrix constituting the solution point. The sources are visualized in Figure 4.

## 4. Discussion

In this work, we studied the neural correlates in a group of young, healthy volunteers derived by the gustatory ERPs in response to sodium chloride, potassium chloride, and a 50% mixture of the two and their perceptual properties, employing a cutting-edge configuration system [31,32]. No other similar works of this typology are available. The main results of the study can be summarized in the following concepts.

Although chemically different, the perceptual ratings of the three stimuli were indistinguishable for intensity, saltiness, and pleasantness.

The time course of the three gustatory processes, from the initial 63 ms to 534 ms, exhibited similarities in scalp topographies, although the chemicals were different, as indicated by the results of the microstates segmentation (Figure 2). The main topographical differences among the three conditions appeared in the late part of the epoch, a time range classically associated with the endogenous phase of perception, i.e., the personal valence of the stimulus condition.

The sources of these microstates shared brain activities located in close areas of the right frontal gyrus pars opercularis.

### 4.1. Palatability of the Potassium Chloride

The participant group rated the solution with potassium chloride as having the same valence as the fifty–fifty mixture solution and the pure NaCl solution. Although some studies have reported that a significant number of people find KCl extremely unpleasant, often describing a bitter/sour aftertaste [17,45,46] and metallic flavor [47], our psychophysics results indicated that the mixture condition was rated at the same hedonic level as the pure potassium chloride and pure sodium chloride solutions.

Our findings indicate that KCl, NaCl, and a fifty–fifty mixture of the two (N50K50) can be used interchangeably in terms of pleasantness, without significantly affecting the palatability of taste products. This conclusion is based on a sample population of young, healthy individuals, and provides critical insights into the potential applications of potassium chloride (KCl) as a sodium chloride (NaCl) substitute in dietary interventions aimed at reducing excessive sodium intake. This aligns with public health initiatives targeting sodium reduction to mitigate hypertension and associated cardiovascular risks both in America [48] and more recently also in Europe [11].

### 4.2. Scalp Topography and Brain Sources of Salty-Taste

The multichannel neurophysiological approach with the estimation of the source localization allowed us to see if stimulation of the oral cavity with different salt conditions also corresponded to different/similar brain networks [28,36].

We found eleven (11) microstate topographies that described the process of perception of sodium chloride, potassium chloride, and the mixture of them, nine of them independent. Six of the eleven, Map 2, Map 3, and Map 5, which also includes Map 4 and Map 6 and Map 7, were common to the three conditions and also aligned in the time range from 64 to 535 ms, indicating that the three conditions shared a basic spatiotemporal neuronal path of the gustatory process.

Moreover, five maps were unique for each condition.

Interestingly, for pure KCl, the first initial milliseconds (ca. 63 ms) of the time course were occupied by a unique map, Map 1, which is also confirmed by the GEV of Map 1 at the single-subject level, which averaged 19% of the GEV on the group, validating the importance of this map. The classical theory of ERPs explains this early stage of the signal as being influenced by exogenous characteristics of the stimulus (exogenous phase of ERP signals), or, in other words, the ERP signal is determined by the physical properties of the stimulus, such as temperature, intensity, or, as in this case, chemical characteristics [49]. The presence of Map 1 in KCl, then, is likely related to the entirely different characteristics that KCl has compared to the NaCl and the mixture. A promising direction for future research could be to explore the potential correlation between Map 1 in the KCl condition and subjective ratings of saltiness and intensity for solutions with different KCl concentrations. By designing experiments that systematically vary KCl concentrations while collecting both neural and subjective data, future studies could provide deeper insights into how the brain processes the physical characteristics of salt substitutes and their relationship to perceptual experiences.

Maps are best interpreted in terms of underlying sources that generate the maps. The estimated source of M1 is located in the right centro-posterior insula/Rolandic operculum, which has been identified as the gustatory cortex [50,51,52] However, due to the heterogeneity of the insular cortex, these areas are also viewed as hubs for integrating information related to sensory perception and the novelty of experiences [5,53,54,55] and more interesting in encoding the familiarization of novel tastants, as well as in the acquisition, retention, and extinction of aversive taste memories [56,57,58].

We can speculate that the response to KCl in the insula may also apply to this situation. Our study included healthy young adults with no history of issues related to salt intake, indicating that they were unfamiliar with KCl. Therefore, it is reasonable to suggest, based on the evidence reviewed, that the initial microstate observed in the KCl condition could be related to the process of encoding and familiarizing oneself with this new taste.

It is also important to mention that that potassium plays a role in the transduction mechanisms underlying the perception of sour and bitter stimuli, primarily at the receptor level. This involvement has been attributed to its influence on ion channels and receptor signaling pathways, contributing to the overall neural response to these taste modalities [59]. In our study, while we did not specifically investigate potassium’s role in the transduction mechanisms, the neural activations specifically observed for KCl may reflect downstream effects of receptor-level events influenced by potassium dynamics. Future studies could further explore the interplay between receptor-level mechanisms and central processing to deepen our understanding of taste perception.

Apart from Map 1 for KCl, they were all situated at the end of the epoch in a time frame that is classically interpreted as linked to the subjective stimulus significance [49,60,61], increasing appetitive or aversive motivational systems. From this perspective, late maps can be considered as encoding the significance content of the three taste conditions, with the brain able to fine-distance across the chemicals, although the differences in pleasantness and saltiness are imperceptible. The late epoch seems different in each condition and is represented by three distinct topographies. Interestingly, all three maps, Map 8, Map 10, and Map 11, have one source in a common brain region, the operculum (Op), specifically Map 8 in POp, Map 10 in FPOp, and MAP 11 in FOp. Studies have demonstrated that Op plays a crucial role in gustatory function, serving as a critical component in the brain’s processing of taste (Iannilli-neuroimaging and other papers). According to [62] Chikazoe et al., the operculum and the mid-insula exhibit distinct patterns of neural activity in response to basic taste stimuli such as sweet, salty, bitter, and sour, making the Rolandic operculum, a central region inside the operculum, the most likely candidate for the primary gustatory cortex, but also auditory, pain, and vestibular primary areas [54]. Mazzola et al. [63] provided further evidence through direct electrical stimulation studies. They reported that gustatory sensations could be evoked reliably in the posterior operculum, overlapping with areas responsible for oral somatosensory inputs. This overlap underscores the integrative role of the posterior operculum in creating a unified perception of flavor, combining taste with oral somatosensory inputs.

Additionally, Mălîia et al. [64] emphasized the functional connectivity between the operculum and insula. Their findings highlight the posterior operculum’s involvement in a broader network that integrates sensory inputs from multiple modalities, reinforcing its role in the complex sensory experience of taste.

These findings collectively suggest that the operculum is a multimodal cortex with widespread connectivity. Its multiple-site cortex devoted to gustatory functions is clear, but this does not exclude that this structure can act as a high-level hedonic function colocalized with low-level sensory representation, similar to what Anderson and colleagues [62] have theorized.

Finally, other frontal areas, such as the MTFpG and IFGtrp, are explained by the functionality of high-order-association cortical areas, consistent with other evidence that locates their affective and hedonic properties of taste and flavors [65]; the understanding of the neural responses can help to address potential concerns about the taste or acceptance of KCl, making it easier to introduce as a sodium substitute in everyday diets.

### 4.3. Study Limitations and Future Research

This study was conducted on a healthy, young population, which means that the conclusions are limited to this age group. Given the well-documented decline in taste function with age, we cannot generalize our findings to older populations, nor can we infer how age-related changes might affect the underlying neural processes observed at the brain level.

Additionally, our analysis relied on a physical model to estimate the dipoles, incorporating spatiotemporal constraints to enhance the stability and reliability of the results. While this approach has been widely and successfully applied, determining the exact number of dipoles remains a challenge, which may introduce variability in the fitted solutions.

Lastly, we did not assess participants’ familiarity with the stimuli or their personal significance, which may limit some of the deductions we have reported. Future studies should consider incorporating these factors to better understand their potential impact on neural and behavioral outcomes.

Future research should focus on extending these findings to diverse population groups, including older adults and those with pre-existing taste impairments, to ensure broad applicability. Moreover, longitudinal studies assessing the health outcomes of long-term KCl use as a salt substitute could provide further evidence to support widespread adoption in dietary guidelines.

## 5. Conclusions

In conclusion, our study contributes to a more comprehensive understanding of the neural mechanisms involved in salt taste perception and offers new insights into the potential for KCl as a healthier alternative to NaCl. By focusing on brain activity, we tried to clarify the role of specific neural circuits in the perception of salty taste, which may ultimately inform strategies for dietary interventions aimed at reducing sodium intake and improving public health outcomes.

Applications of this research extend beyond public health into food technology and policy-making. Food manufacturers can leverage these findings to develop reduced-sodium products tailored to specific demographic groups, considering that younger populations may find KCl-based products more palatable. Additionally, policy-makers could advocate for KCl incorporation in processed foods as a regulatory standard to reduce sodium levels nationwide.

## Figures and Tables

**Table 1:** For each condition listed in the table, we report the corresponding unique maps, the relative Talairach coordinates of the sources, the names of the areas described in the Mai atlas [42], and the source localization points in the matrix of distributed sources used for estimation.

Condition	Map	Talairach (mm)			Brain Area	Source Point
		x	y	z		
KCl	Map 1	40	−20	16	Insula (Ic)/Rolandic Operculum (ROp)	RPS38
	Map 8	−48	38	2	Inferior Frontal Gyrus triangular part (IFGtrp)	LAI371
		4	58	−2	Medial Transverse Frontopolar Gyrus (MTFpG)	RAI 431
		43	−27	22	Parietal Operculum (POp)	RPS 171
Mixture	Map 9	50	−60	19	Superior Temporal Gyrus (STG)	RPS144
		−48	31	14	Inferior Frontal Gyrus triangular part (IFGtrp)/Medial Frontal Gyrus (MFG)	LAS624
	Map 10	57	−16	22	Frontoparietal Operculum (FPOp)	RAS52
		24	57	3	Medial Transverse Frontopolar Gyrus (MTFpG)	RAI265
		−48	38	2	Inferior Frontal Gyrus triangular part (IFGtrp)	LAI 371
NaCl	Map 11	51	5	16	Frontal Operculum (Fop)	RAS33
		−45	2	42	Medial Frontal Gyrus (MFG)	LAS637

## Data Availability

The data supporting the findings of this study are available from the corresponding author upon reasonable request. Access to the data may be subject to restrictions, such as the need for institutional approval or confidentiality agreements.

## Supplementary Materials

The following supporting information can be downloaded at: https://www.mdpi.com/article/10.3390/life15020207/s1, Figure S1. Butterfly plot displays the gustatory event-related potentials (gERPs) for three taste conditions: KCL (K), NaCl (N), and a mixture of 50% NaCl and 50% KCl (N50K50), recorded using a 128-channel EEG. The onset of the stimulus is marked at the zero point on the x-axis (time axis) and is indicated by a red arrow.
