# Supplementary material for: The Neural Basis of Salt Perception: A Focus on Potassium Chloride as a Sodium Alternative
Source: Life (Basel). 2025 Jan 30;15(2):207. doi: 10.3390/life15020207 (PMC11856358; doi:10.3390/life15020207)

Supplementary Material

manuscript title:

*The Neural Basis of Salt Perception: A Focus on Potassium Chloride as a Sodium Alternative*

E Iannilli, R Fürer, A Welge-Lüssen and T Hummel

life - 3379269

**Figure S1.** Butterfly plot displays the gustatory event-related potentials (gERPs) for three taste conditions: KCL (K), NaCl (N), and a mixture of 50% NaCl and 50% KCl (N50K50), recorded using a 128-channel EEG. The onset of the stimulus is marked at the zero point on the x-axis (time axis) and is indicated by a red arrow.

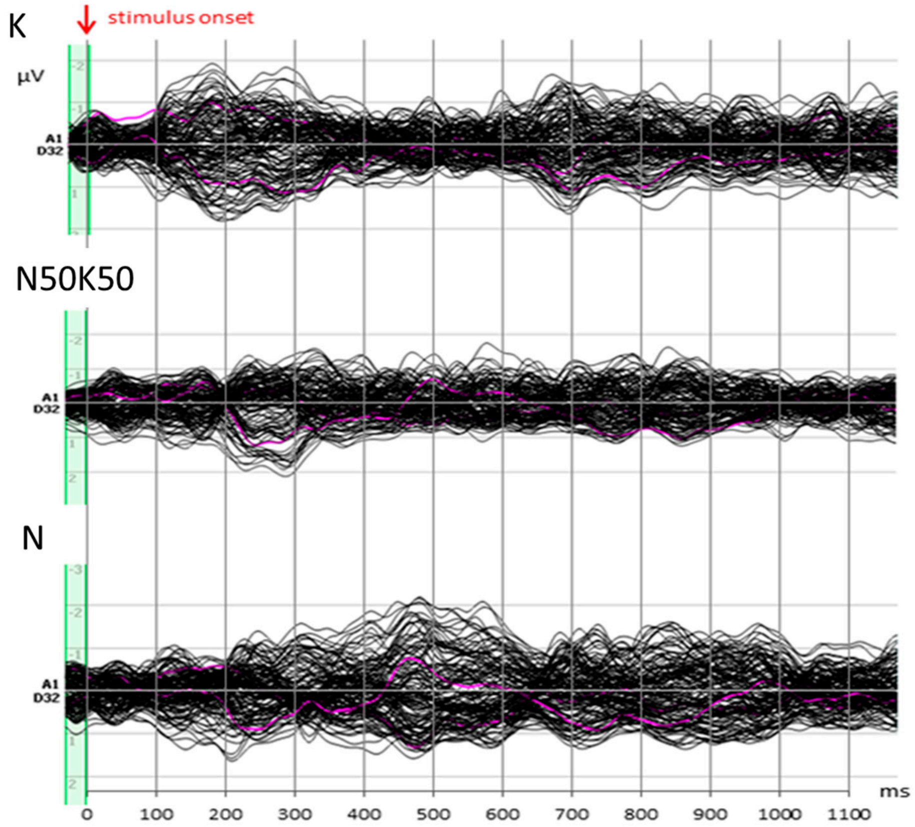

Supplement: Supplementary file 1 [file life-15-00207-s001.zip › life-3379269-supplementary.pdf]
